# Caregivers’ Experiences of Family-Based Trauma-Informed Interventions for Children with Prenatal Alcohol Exposure

**DOI:** 10.1007/s40653-026-00963-7

**Published:** 2026-08-14

**Authors:** Ellie Shepherd, Jack Purrington, Buket Kara

**Affiliations:** 1 Division of Health Research, Lancaster University, Lancaster, United Kingdom; 2 Chrysalis Associates, Sheffield, United Kingdom

**Keywords:** Prenatal alcohol exposure, Trauma-informed, Family-based, Trauma, Fetal alcohol spectrum disorder, Therapy

## Abstract

Children prenatally exposed to alcohol often face a multitude of difficulties such as mental health difficulties and behavioural and cognitive challenges. A high percentage of children with prenatal alcohol exposure (PAE) have also experienced traumatic life events. Research suggests that interventions can help reduce the difficulties faced after trauma, particularly when involving the caregiver and child. Due to the cognitive difficulties often experienced by children with PAE, it is unclear whether typical trauma-informed interventions can be effectively used with this population. This study aimed to explore the experiences of caregivers whose children with PAE participated in a family-based, trauma-informed intervention. Eleven caregivers (10 adoptive, one foster) were recruited via therapeutic services in the UK and an established FASD mailing list. Caregivers were caring for a total of 12 children with PAE aged 8 to 18. A variety of interventions were received, including Theraplay, Dyadic Developmental Psychotherapy, Cognitive Behavioural Therapy, Sandplay Therapy, therapeutic parenting sessions and sensory therapies. These were accessed for varying lengths of times and in different combinations. Reflexive thematic analysis was employed to explore semi-structured interview data, with particular focus on perceived benefits and challenges, children’s ability to engage in therapy, potential outcomes and factors influencing change. This generated five interrelated themes: (1) Enduring the therapeutic journey to survive, (2) Engagement as fluid and negotiable, (3) The therapeutic relationship is the active ingredient, (4) “There’s no magic button”: supported caregivers are the agents of change, (5) Redefining success. Family-based therapy was positioned as a relational, effortful, and ongoing process that supports families to live with enduring complexity. Therapeutic change was primarily located within caregivers’ development of understanding, responsiveness and ability to adapt the caregiving environment, as opposed to within the child. Engagement emerged as negotiated and developmentally contingent, with the therapeutic relationship as a central component. Therapy was shaped by the perceived permanence of PAE and trauma and by wider systemic constraints. The findings support family-based, flexible therapeutic approaches for children with PAE and trauma that prioritise relational safety and caregiver understanding. Early sensory and play-based interventions, integration of therapeutic models, and caregiver-only sessions may enhance effectiveness.

## Introduction

Prenatal alcohol exposure (PAE) is highly prevalent in the UK, with an estimated 40% of mothers self-reporting alcohol use during pregnancy (Popova et al., 2017). PAE can cause effects on fetal and neurodevelopmental outcomes including altered brain development, congenital anomalies, and changes in cognitive or physical functioning (Lebel et al., 2011), resulting in lifelong support needs. Fetal Alcohol Spectrum Disorder (FASD) is the neurodevelopmental diagnosis often given to capture the broad range of effects associated with PAE (Jacobson et al., 2021); however, not everyone with PAE will receive a diagnosis. Whilst the effects of PAE can vary depending on timing, duration, quantity and frequency of alcohol consumption during pregnancy (Davis et al., 2013), research has documented that children diagnosed with FASD face a multitude of challenges including cognitive, learning, behavioural, social and emotional difficulties (Khoury et al., 2018; Mattson et al., 2019). Furthermore, an estimated 90% of those diagnosed with FASD experience mental health difficulties in their lifetime (Pei et al., 2011).

In addition to the impact on the individual, PAE and FASD can also negatively affect families’ and caregivers’ quality of life (Reid & Moritz, 2019). Caregivers report needing greater support, often because children struggle to respond to typical parenting approaches (Chamberlain et al., 2017). Furthermore, underlying difficulties with physiological and emotional regulation place children with PAE and FASD at heightened risk for developing insecure attachment relationships (O’Connor et al., 2002; Reid & Petrenko, 2018). Research has highlighted the importance of the family environment, particularly stability and positive parental involvement, in supporting children with an FASD diagnosis (Jacobson et al., 2004; Streissguth et al., 2004). Consistent and nurturing relationships with caregivers appear to strengthen resilience and improve outcomes (Kully-Martens et al., 2023). Additionally, providing caregivers with information about FASD and associated challenges has been shown to improve parenting confidence and capacity (Davis et al., 2017). As such, multitiered interventions including both the child and caregiving system may be necessary for meaningful and sustained improvements.

Compounding the neurodevelopmental effects of PAE and FASD is the prominent level of trauma and adversity among those affected. Research shows that children with PAE are more likely to experience adverse childhood experiences (ACEs) (Kautz-Turnbull et al., 2023), trauma (Price et al., 2017), and out-of-home placements (Tenenbaum et al., 2020) compared to those without PAE. A recent systematic review suggests that individuals diagnosed with FASD experience an average of four ACEs (Rockhold et al., 2025). Such experiences may be associated with further neurodevelopmental changes (Price et al., 2017), exacerbating the existing difficulties associated with PAE. This intersection between neurodevelopmental vulnerability and postnatal adversity highlights the importance of trauma-informed approaches for this population.

In the broader trauma literature, numerous therapeutic approaches appear to effectively alleviate common difficulties following ACEs and trauma in non-PAE children (Leenarts et al., 2013; Lorenc et al., 2020). A recent systematic review highlighted that family-based approaches, such as Developmental Dyadic Psychotherapy (Hughes et al., 2019) and Theraplay (Booth & Jernberg, 2009) show promise in supporting adoptive families (Purrington et al., 2024). Early evidence suggests that integrating such approaches may lead to improvements in attachment, emotional regulation and trauma-related symptoms for children with PAE (Purrington, Goodall et al., 2025a, b). Furthermore, preliminary research has suggested that a multi-component family-based intervention for children with FASD is feasible, acceptable and can improve outcomes related to attachment and psychosocial distress (Reid et al., 2017). However, research remains sparse and relatively few therapeutic approaches meaningfully incorporate both caregiver and child.

Despite increasing awareness of PAE and its consequences, UK clinical and service-delivery guidance for supporting individuals with PAE remains limited. Recent guidelines from the National Institute of Health and Care Excellence (NICE, 2022) provide a framework for diagnosing, assessing, preventing and managing FASD; however, they lack direction on intervention or support and fail to acknowledge PAE more broadly. Although FASD is a lifelong neurodevelopmental condition, early diagnosis and targeted intervention can ameliorate some commonly faced challenges (Kodituwakku & Kodituwakku, 2011). Recent reviews have demonstrated promising results with interventions for children with PAE and FASD and their caregivers (Flannigan et al., 2020; Ordenewitz et al., 2021; Reid et al., 2015), though most target single areas of difficulty such as attachment, executive functioning or social skills, rather than the multifaceted needs of this population. Effective interventions likely require a more holistic, trauma-informed, systems-based approach involving both the child and their caregivers (Kodituwakku, 2010).

It remains unclear whether children with PAE, who often present with significant cognitive, attentional and executive functioning challenges (Davis et al., 2013), can effectively engage with and benefit from conventional trauma-based therapies. Whilst several studies have explored the lived experiences of individuals with an FASD diagnosis and their families, there remains a lack of research examining families’ experiences of accessing family-based, trauma-informed interventions for children with FASD, and PAE more broadly (Domeij et al., 2018).

Given the high prevalence of children with PAE in the UK, alongside the high level of ACEs and challenges faced by affected children and their families, it seems crucial to understand which interventions are effective and how they are experienced. To address this gap, the present study aims to explore the experiences of caregivers whose children with PAE participated in a family-based, trauma-informed intervention. By exploring caregivers’ perspectives, this study aims to identify the perceived benefits and challenges, children’s ability to engage in therapy, potential outcomes and factors influencing change.

## Methods

### Design

A qualitative approach was determined appropriate to explore participants’ experiences of family-based trauma-informed interventions for PAE. Reflexive Thematic Analysis (RTA) (Braun et al., 2023) was chosen for its flexibility and suitability in addressing the studies’ multiple research aims, including understanding experiences, identifying benefits and challenges, potential outcomes and factors influencing change (Braun & Clarke, 2013). RTA allows for identifying both individual meaning-making and shared patterns of experience across participants, making it well-suited in understanding both the subjective experience and the practical implications of interventions (Braun & Clarke, 2021). The Reflexive Thematic Analysis Guidelines informed analysis and reporting (Braun & Clarke, 2024).

### Participants

A combination of purposive and convenience sampling was used. Three UK-based therapeutic services supporting children with trauma and attachment difficulties distributed recruitment materials to eligible families. Furthermore, recruitment materials were distributed to all members of an FASD mailing list created by the [masked for blind review]. Participants were eligible if they were a caregiver of a child with PAE or FASD who had engaged in family-based, trauma-informed therapy with their child within the past three years. Due to challenges in accessing formal FASD diagnoses and evidence of PAE, eligibility criteria required only caregiver awareness of alcohol use during pregnancy. Therapies were considered family-based if they involved the child and at least one other family member in at least half of the sessions. Therapies were considered trauma-informed if they aimed to create emotional and physical safety, strengthen relationships, and support healing through understanding the impact of trauma on behaviour, emotions, and family dynamics (SAMSHA, 2014). Therapies were required to reduce trauma-related symptomology, as opposed to singular deficits such as social difficulties. Suggested therapies included DDP and Theraplay (commonly used trauma therapies which involve the wider system); however, other therapies were not excluded.

An ‘information power’ approach (Malterud et al., 2016) guided sample size, whereby consideration was given to the broadness of the study aims, sample specificity, established theory, quality of dialogue, and analysis strategy. These factors were reviewed throughout interviews until the researcher and their supervisor felt sufficient information power was reached.

Fifteen caregivers expressed interest in the study; however, one did not meet inclusion criteria and three did not follow-up on invitations to participate. A total of 11 participants took part in the study, including 10 adoptive parents from nine families, and a foster carer. Two participants were in a relationship and were interviewed together. Participants included six females and five males aged 40 to 57 who were caring for a total of 17 children. One caregiver was Irish and the remaining White British, with all families residing in the UK.

In cases where caregivers cared for multiple eligible children, they were given the option to focus on either one child or reflect on experiences from both. Participants discussed a total of 12 children with PAE aged 8 to 18 (*M =* 15.5). Six children were male, five were female, and one was female at birth but identified as male at the time of the interview. One child was Mixed White and the remainder White British. A total of six children had a formal diagnosis of FASD; however, the severity of alcohol exposure was difficult to confirm. Age at current placement ranged from birth to five years. Full participant demographics are presented in Table 1.

**Table 1:** Participant Demographics

	Category	*n*	
Caregiver Characteristics			
Age (years)	40–44	3	
	45–49	2	
	50–54	4	
	55–59	2	
Gender	Female	6	
	Male	5	
Ethnicity	White British	10	
	White Irish	1	
Marital status	Married	8	
	Cohabiting	1	
	Separated/Divorced	2	
Number of children in household	1	5	
	2	6	
Highest educational qualification	GCSEs or equivalent	2	
	A-levels or equivalent	2	
	Undergraduate degree		5
	Postgraduate degree	2	
Employment status	Employed full-time	4	
	Employed part-time	4	
	Self-employed	2	
	Retired	1	
Household income (£)	£25,000-£34,999	2	
	£35,000-£44,999	1	
	£55,000-£64,999	1	
	£65,000-£74,999	3	
	£85,000–£99,999	2	
	Over £100,000	2	
Child Characteristics			
Age (years)	8–10	1	
	11–13	4	
	14–16	6	
	17–18	1	
Gender	Female	6	
	Male	5	
	Other	1	
Placement type	Adopted	11	
	Fostered	1	
Age at placement	0–11 months	3	
	1–2 years	5	
	3–4 years	0	
	5–6 years	3	
	Not disclosed	1	
Age at start of therapy (years)	7–9	6	
	10–12	2	
	13–15	3	
	Not disclosed	1	
Ethnicity	White British	10	
	Mixed British	1	
	Not disclosed	1	
Diagnoses	FASD	6	
	ADHD	6	
	Autism	2	
	Attachment disorder	1	
	Developmental trauma	1	
	Executive function disorder	1	
	Sensory processing disorder	1	

Of the included families, seven children received a combination of Theraplay, DDP and parenting sessions. Others received Theraplay with parenting sessions, DDP alone, sandplay therapy, or cognitive behavioural therapy with partial parental involvement. Some also accessed sensory therapy and Eye Movement Desensitisation Reprocessing (EMDR). Length of therapy ranged from six months to four years, with four children still in therapy at the time of interview. Full intervention details are presented in Table 2.

**Table 2:** Intervention Characteristics

Intervention Characteristic	Category	*n*
Intervention type	Theraplay & parenting sessions	1
	DDP	2
	Integrated Theraplay, DDP, parenting sessions	7
	Sandplay therapy	1
	CBT	1
Previous therapeutic input	BUSS	3
	EMDR	2
	Art therapy	1
	Nonviolent resistance training	2
	Psychologist support	1
Length of therapy	Less than 1 year	4
	1–2 years	2
	3–4 years	5
	5 years +	1

### Ethics

Ethical approval was gained from the Faculty of Health and Medicine Research Ethics Committee at Lancaster University (Reference: FHM-2025-4498-SA-2) in February 2025. Both written and verbal consent was obtained from participants. Given the likelihood of previous distressing experiences in this population, participants were reminded to share only what felt comfortable. A distress protocol was established to guide the researcher’s response should any participant exhibit signs of distress; however, no participants became distressed during the interviews. Personal information was redacted from transcripts, and participant identifiers were used for anonymity.

### Procedure

Therapeutic services for children with trauma and attachment difficulties across the UK were approached to support recruitment. Five services were contacted, and three of these distributed recruitment materials to eligible families. Materials were also distributed using an established FASD research mailing list. Interested individuals contacted the researcher to register their interest. They were then sent a link to an information sheet, consent form and demographic questionnaire.

Semi-structured interviews were conducted between April and December 2025. Participants were given the option to participate in interviews online, in-person or via phone; however, all participants opted to use the videoconferencing software *Microsoft Teams.* Interviews were recorded using this software and were guided by an interview schedule consisting of open-ended questions to allow the exploration of participants experiences of the intervention. Questions were co-produced with two experts by experience and explored contextual information, experiences of therapeutic input, helpful and unhelpful aspects of therapy, differences noticed within the child and family, and factors influencing change. Interviews ranged in duration from 29 to 68 min (*M* = 50.4) and were transcribed verbatim by the main researcher.

### Data Analysis

Data were analysed using RTA following the guidance of Braun et al. (2023). Data was predominantly analysed by the first author, however additional authors provided feedback to support in refining final themes. Inductive approaches were employed whereby the analysis was predominantly data-driven; however the researcher’s awareness of existing theories likely influenced interpretations. A latent orientation consistent with constructionist epistemology guided the analysis to explore underlying meanings and assumptions within participants’ accounts.

Interview transcripts were read and re-read, with initial notes made to support familiarisation and early analytic insights. Transcribed data were then coded line by line to generate initial flexible codes. As most data came from individual interviews, the dyadic interview was treated largely as a single dataset. The two participants often co-constructed narratives, whereby one would start answering a question and the other would then provide additional insights; therefore, this interview was mostly coded and analysed as one dataset. However, where disagreements occurred, perspectives were considered separately in relation to the research questions, and both perspectives were included within the analysis.

Codes were examined for patterns of shared meaning and organised into initial themes, which were reviewed, refined and developed, including the generation of sub-themes where appropriate. Themes were then defined and named to capture their central organising concept and relevance to the research questions.

### Reflexivity

RTA emphasises the influence of the researcher’s background, experiences, theoretical commitments and social position on how data is interpreted and analysed (Braun & Clarke, 2019). The first author, who led the data analysis and interpretation process, was a trainee clinical psychologist with professional experience of working therapeutically with children affected by PAE. They occupied a position that inevitably shaped the analytic process. Within RTA, this intersubjectivity is viewed as an inherent and valuable aspect of qualitative enquiry. The first author’s clinical background provided familiarity with the challenges faced by families and services, as well as an awareness of dominant discourses related to trauma, attachment and neurodevelopment.

Reflexivity was embedded throughout the research process. A reflexive journal was maintained to document assumptions, expectations, analytic decisions, and reflections during interviews and analysis. Regular reflective discussions with the co-authors considered how prior knowledge and experiences may have affected sensitivity to certain meanings. These reflexive practices aimed to enhance transparency, rigour, and coherence.

## Results

The reflexive thematic analysis generated five interrelated themes capturing caregivers’ constructions of family-based therapy for children with PAE and trauma (see Table 3). Across accounts, therapy was positioned not as a process of curing difficulties, but as a relational and adaptive process that enabled families to live with the enduring effects of both PAE and trauma. Caregivers consistently described how neurodevelopmental differences associated with PAE, alongside experiences of trauma and attachment disruption, shaped expectations of therapy, children’s engagement, and understandings of therapeutic success. Therapeutic change was primarily located within caregivers’ development of understanding, responsiveness and ability to adapt the caregiving environment, rather than within the child alone.

**Table 3:** Themes generated from reflexive thematic analysis

Theme	Sub-themes
Enduring the therapeutic journey to survive	• Accessing services: a matter of chance
	• Emotionally exhausting and challenging
	• It’s a slow journey, but worthwhile
Engagement as fluid and negotiable	• Engagement looks different
	• Timing matters
	• Therapeutic fit: Play first, talk later
The therapeutic relationship is the active ingredient	• Trust and safety take time
	• Therapist over therapy
	• Validation as an intervention
“There’s no magic button”: Supported caregivers are the agents of change	• Developing an understanding of trauma responses
	• Learning what works: adjusting parenting approaches
	• Beyond adjunct: caregivers driving therapy outcomes
Redefining success	• Letting go of cure-oriented expectations
	• Success as understanding and responsiveness
	• :Living better with ongoing complexity

### Theme 1: Enduring the Therapeutic Journey to Survive

Participants highlighted the importance of accessing support and services; however, this was frequently framed as a matter of luck rather than entitlement. When accessed, family-based therapy was described as emotionally demanding and requiring endurance over time, yet ultimately worthwhile.

#### Accessing Services: A Matter of Chance

Caregivers described difficulties accessing therapeutic support, with access often framed as a matter of luck. Very few children had received FASD diagnoses, and caregivers of those who had felt “very fortunate”, noting that “nine other families won’t get it” (Participant 9). Diagnosis sometimes functioned as a gateway to understanding and support within systems, particularly where trauma had previously been dismissed:

So having that foetal alcohol syndrome diagnosis just means that school are a bit like, oh, so there is a physical disability, you know, there is. And [child]’s always been registered disabled, but it’s almost like trauma doesn’t really count. (Participant 9).

However, diagnosis did not always lead to support. Caregivers described encounters with services and professionals who lacked understanding, with one recalling being told “it’s brain damage, nothing we can do, good luck” (Participant 3), and another stating “you get really just clueless professionals sadly, sometimes who make really bad decisions because they don’t have the knowledge” (Participant 5). Therapeutic provisions were described as unequal and “terrifying” (Participant 6), resulting in participants feeling “incredibly lucky” (Participant 8) when receiving them. Caregivers described having to “fight for everything” (Participant 3), adding to the existing exhaustion. Even once support was accessed, there remained uncertainty about future support due to funding cuts and changes, with caregivers finding this “worrying” (Participant 3) and reflecting that “as a parent, it gives you a lot of anxiety” (Participant 8).

For one family, diagnosis led to practical support, including a funded personal assistant. This was considered “vital” for coping, with the participant suggesting they’d be “in a ball on the ground if I wasn’t getting a wee breather” (Participant 10).

#### Emotionally Exhausting and Challenging

In addition to the challenges of navigating systems, caregiver accounts highlighted the intense challenges of caring for children with PAE and trauma, including aggression, attachment difficulties and emotional dysregulation. Therapy was often sought at “crisis point” (Participant 5; 8), “breaking point” (Participant 7; 9) or when caregivers were “at my wit’s end” (Participant 6). Within this context, therapy itself was described as an additional demand requiring emotional and practical effort. Families faced barriers such as extensive travel and time off work, with one caregiver describing this as “frazzly” (Participant 5). Sessions themselves were experienced as emotionally demanding, “hard work” (Participant 1B), and “very challenging” (Participant 8), requiring sustained emotional availability and tolerance of uncertainty. One participant explained, “it’s easy to turn up, but to be actually fully emotionally available for your kids, week on week irrespective of how you’re feeling in a very time limited way on the days is exhausting” (Participant 2).

#### It’s a Slow Journey, but Worthwhile

Therapy was frequently described using journey metaphors, highlighting it’s incremental and uncertain nature. Caregivers described an initial “settling period” (Participant 8), characterised by confusion and doubt. One caregiver recalled wondering “what is this? How is this even gonna do anything?” (Participant 1 A). This was followed by a “long process” (Participant 8) where progress was “slow” (Participant 2) and children “can’t do too much too soon” (Participant 5). This could feel frustrating when families were seeking immediate relief:

It took a very long time to build those relationships. And I think that the beginning part, I think we’d be very honest to say it was quite frustrating at the start because like we’re in crisis, we need answers, we need help and it was all very, very gentle. (Participant 8)

Despite this, caregivers persisted due to a sense of responsibility, believing they “had to do it” (Participant 3). Caregivers also believed in its necessity, with one caregiver stating “it’s needed. You know, it’s not going to get better on its own” (Participant 6).

Despite the journey being slow and challenging, therapy was widely described as “worthwhile” (Participant 1B), with one participant reflecting “even the hard bits have been worth it” (Participant 6).

Successful therapeutic experiences were often described as unfolding over years with caregivers acknowledging they were “constantly learning” (Participant 1 A), even after formal interventions had ended. Therapy was therefore constructed as requiring patience and perseverance.

Taken together, these subthemes position therapy as occurring within a context already shaped by the cumulative demands of caring for children affected by PAE and trauma. Accessing support often required extensive advocacy and persistence, whilst therapeutic engagement itself represented an additional emotional burden. Caregivers therefore approached therapy from positions of exhaustion and crisis, which subsequently shaped both their expectations of intervention and their definitions of therapeutic success.

### Theme 2. Engagement as Fluid and Negotiable

Engagement in family-based therapy was depicted as changeable, context-dependent and actively negotiated over time, often looking differently to expected. Caregiver accounts emphasised the impact of PAE and trauma, developmental readiness, motivation, and therapeutic fit.

####  Engagement Looks Different

Caregivers described their children’s engagement in therapy as changeable, interpreting engagement more broadly than attendance or verbal participation. Children were described as engaging through listening, observation, or physical responses even when appearing disengaged. One caregiver stated “a lot of people would look at it and say she’s not engaging with it, but if you knew her you would know that she is engaging with it” (Participant 5), whilst another noted “It’d be the adults talking about something with him listening, even though he didn’t act like he was listening” (Participant 6).

Children’s engagement often fluctuated week by week, with one caregiver describing “good days” where it felt like progress and other days where “you didn’t get anything back” (Participant 2). Some children would only engage “on his terms” (Participant 1 A), emphasising the need for negotiation through flexibility and adaptability. Awareness of the reasons behind fluctuating engagement supported adaptations, such as the child’s need to be in control, feelings of vulnerability, or challenges occurring outside of therapy.

One family made accommodations by allowing the child to not directly participate in the therapy, instead using the caregivers as mediators. In this case, the child still appeared to value the therapeutic relationship, with the caregiver noting “he’ll sometimes say ‘oh, have you asked [therapist] that?’ so I do think that positive link is there. It’s a bit like I want it to be there, I just don’t want to have to do it myself” (Participant 6).

#### Timing Matters

Caregivers emphasised that engagement could not be forced and was shaped by developmental transitions, family circumstances, and emotional readiness. Adolescence was described as a period where engagement could increase or decrease as children became more reflective. One caregiver wondered “if he’s at an age where he actually wants the help now” (Participant 3). Transitions also led to new challenges, with one caregiver reflecting “as she develops and as she is growing into an adult, we’re now facing very different challenges and crisis moments” (Participant 8).

Disengagement was often framed as temporary and situational, with therapy potentially becoming relevant again when older or at “the right time in their life” (Participant 6). External stressors also impacted on engagement, such as divorce, bereavements and school difficulties. Therapy was often considered ineffective when accessed during periods of acute distress, with one caregiver suggesting support was more useful if they “wait until [child]’s in a really good place” (Participant 9). Some caregivers felt therapy had been offered too late, with one caregiver describing their child as “too far gone” (Participant 7) and another stating things would have been “a heck of a lot smoother” (Participant 4) if therapy had been offered sooner.

#### Therapeutic Fit: Play First, Talk Later

Engagement was shaped by perceived fit between the therapeutic approach and the child’s developmental, emotional and neurodevelopmental profile, with engagement more likely when therapy aligned with communication styles and interests. When disengagement occurred, this was attributed to a lack of therapeutic fit rather than child resistance.

Play-based and sensory approaches were constructed as more accessible for younger children or those with significant emotional dysregulation, enabling participation without verbal demands. These approaches were described as being on the child’s “wavelength” (Participant 5) and were often portrayed as a preparatory stage for later verbal therapy. Caregivers described play as a means of improving attachment quality and building a “safe space to talk” (Participant 2). As children developed, caregivers described a shift in what constituted a good therapeutic fit, with play-based approaches becoming “childish” (Participant 7) and “less impactful” (Participant 1B). Talking therapy became more suited as children became more able to communicate verbally. One participant noted that “play was really important in terms of building that initial relationship”, but talking therapy was more successful “just because it just goes a bit deeper” (Participant 5). However, talking therapy often still required play, activities or distraction alongside it to support regulation, with one child benefitting from bringing their pet dog into therapy.

Sensory approaches were consistently described as beneficial due to children’s diverse sensory needs, often described as “‘more suited” (Participant 10) and having “quite a big effect” (Participant 1B). Caregivers were able to learn about their child’s sensory needs and support their regulation through gaining “loads of ideas about how we could use play to help her regulate” (Participant 5) and integrating these into daily routines.

Taken together, these sub-themes highlight the fluctuating nature of engagement, positioning it as a negotiated and dynamic process. Caregivers challenged conventional assumptions that engagement is demonstrated primarily through verbal participation, compliance or attendance, instead recognising more subtle forms of involvement. Patterns of engagement were frequently linked to the combined effects of PAE and trauma, including difficulties with emotional regulation, attentional control, trust, sensory processing, and a heightened need for autonomy and control. Consequently, engagement was understood as dependent on timing, therapeutic fit and developmental readiness, requiring flexibility and ongoing adaptation. Caregivers described play-based and sensory approaches as particularly valuable in creating the relational safety and regulatory capacity necessary for later therapeutic work, whilst conventional talking-based approaches were often experienced as inaccessible until these foundations had been established.

### Theme 3: The Therapeutic Relationship is the Active Ingredient

Building on the previous theme, caregivers consistently described the therapeutic relationship as the mechanism through which engagement became possible. Given children’s histories of trauma and disrupted relationships, relational safety was viewed as a prerequisite for therapeutic participation and change.

#### Trust and Safety Take Time

Caregivers frequently described the therapist as a safe and trusted figure for themselves and their children. Relational safety and connection were seen as prerequisites for engagement, particularly given children’s history of relational trauma. Caregivers emphasised the need for their children to “feel safe and feel regulated” in sessions (Participant 5), and the need for “a huge amount of trust” (Participant 10) to engage and communicate. Trust was described as developing slowly, taking “a really long time to build up” (Participant 5) and requiring continuity, familiarity and “early groundwork” (Participant 8). Without this foundation, therapy was ineffective and in some cases distressing. Even once established, it took time for children to feel comfortable talking about their feelings and experiences:

She really has got, like, a strong relationship with the therapist, but it’s still taking her a really long time to be able to talk about anything. I think some people, what they’ve been through, they just need that time. (Participant 5)

#### Therapist Over Therapy

Caregivers often attributed therapeutic effectiveness to the individual therapist rather than the model, with one asking, “is it the therapist or is it the therapy?” (Participant 8). Feeling understood and accepted was central, with one caregiver noting “I think what he likes about this therapist was, he felt the therapist got him” (Participant 1 A). In contrast, perceived misattunement created barriers to engagement, as one caregiver reflected “she definitely wasn’t very open with the therapist because of that barrier of, she just felt that the therapist really just didn’t get her” (Participant 10).

Therapists were valued when they were flexible, responsive and adapted to the family’s changing needs. Caregivers praised their therapist’s ability to “go off plan” (Participant 3) and be “dynamic” (Participant 6) when their child was struggling, whilst also creating a sense of routine and predictability that promoted safety.

Caregivers emphasised the need for the therapist to ‘fit’ the family values, with engagement suffering when this was not the case. For example, one family perceived their therapist as too gentle:

We don’t like airy fairy people we’re not that kind of family. We’d rather have someone that’s a bit straight, tells you how it is, get on with it… whereas [previous therapist] will work for other families but she didn’t for us. (Participant 3)

Another caregiver mentioned the impact of their child’s previous relational trauma on finding the right therapist, stating “the choice of therapist was probably a mistake, like looking back, it was a male therapist, which I don’t think was a good idea.” (Participant 9).

Therapists understanding of FASD and the associated challenges also affected therapeutic impact, enabling validation and promotion of understanding. One caregiver noted “having a therapist who understood the FASD and the impact on the brain and could articulate that and help us and help [child] to understand it… that really was the bit that we benefited from most.” (Participant 8). Another caregiver emphasised that “you can’t overlook it (FASD), you can’t, you know. Because it is almost everything, you know? It kind of top trump’s everything else” (Participant 10).

#### Validation as an Intervention

Caregivers consistently described feeling understood and validated as central to the therapeutic process. Validation reduced feelings of blame, failure and isolation. Through validation, caregivers felt supported and were able to reframe their experiences in less self-critical ways, creating space for reflection, learning and change. One caregiver noted that “having a safe environment for the adults to talk, non-judgmental, giving guidance, giving that space, giving that someone saying, ‘God no wonder you feel like that’ you know, is actually invaluable” (Participant 6).

Children also benefitted from validation, enabling them to feel accepted and understood, with one caregiver reflecting “it gives him somewhere to go and talk to somebody and not be judged and allow him to say whatever he wants to in that room and get it out rather than thinking I’m just barking mad here” (Participant 3).

Formal recognition of FASD was experienced as legitimising and “completely validating” (Participant 9), enabling a shift in how children’s needs were understood and responded to within the family and services. Caregivers described the diagnosis helping them make sense of difficulties, with one reflecting “it doesn’t change anything, but it is useful to have that understanding’” (Participant 6).

Taken together, these sub-themes emphasise the importance of therapeutic safety and illustrate how therapeutic effectiveness can occur through recognition and normalisation of struggles rather than specific models. This positions diagnosis and validation as interventions themselves, and as mechanisms enabling other therapeutic processes to occur.

### Theme 4: “There’s no Magic Button”: Supported Caregivers are the Agents of Change

Therapeutic change was constructed as occurring primarily through caregivers rather than children. Therapy was valued when it helped caregivers develop new understandings of their child, adapt their responses and shape the environment.

#### Developing an Understanding of Trauma Responses

Through therapy, caregivers shifted their understanding of their child, moving away from interpretations of behaviour as intentional and defiant and towards explanations informed by trauma and neurodevelopmental difference. This reframing reduced blame and enabled more compassionate responses. One caregiver realised “this isn’t [child] being difficult or unkind or manipulative, this is [child] struggling” (Participant 9), while another understood “the behaviours that you get, they’re not malice, they’re not designed, they’re just that this is a difficult situation because of the way their brain works” (Participant 2).

Caregivers described learning to read their child’s behaviour as a form of communication, becoming a “detective” (Participant 9). This was helpful given children often could not verbalise or notice their own feelings. Once caregiver reflected “her physical body language tells us how she’s feeling, even if she can’t actually realise what’s going on for herself” (Participant 2).

#### Learning What Works: Adjusting Parenting Approaches

Building on these new understandings, caregivers adapted their parenting approaches to align with their child’s needs, with therapy helping caregivers learn what works for their child. Caregivers recognised that traditional parenting approaches were not effective for their children, with one stating “you can’t revert to traditional parenting. Ever. You can’t” (Participant 2). Caregivers developed child-specific strategies through ongoing therapeutic support, such as scaffolding tasks for their children, helping their child to regulate, anticipating difficulties and responding with more empathy and containment:

We are able to support her in, in, in sort of reminding her of things… So we’re able to sort of say, ‘right, I think you need to use the punch pillow’ or ‘you need to get into your bag’ and, you know and regulate yourself. (Participant 8)

These changes were often presented as subtle yet significant, reducing escalation and reinforcing caregivers’ central role in facilitating change.

There’s no magic button. There’s no therapy that really changes that. We as parents are the agents of change. So if we want to see a change in our kids and their behaviours, it’s something that we have to change and that is difficult. (Participant 2).

#### Beyond Adjunct: Caregivers Driving Therapy Outcomes

Caregiver involvement was positioned as crucial to therapeutic effectiveness. Joint sessions enabled shared understanding, while caregiver only sessions provided emotional support, validation and guidance. Caregivers emphasised “we’ve got to learn as well” (Participant 1B). Where there were multiple individuals caring for a child, caregivers highlighted the importance of them being involved in the therapy sessions to minimise creating a “gap” (Participant 8). Caregiver only sessions were described as “hugely valuable” (Participant 8), and often the “most helpful” (P2), offering space for emotional support, reflection and skill development:

So the parenting sessions were great, erm some, like really good and helpful feedback and tips. And then also I think a lot of it was just having someone to talk things through with and erm just getting like a slightly different perspective on things. (Participant 5).

Caregivers often felt more positive and competent following their sessions, with one caregiver reflecting “she just filled me with positivity and ready to reset and you know, come out of there and try again” (Participant 7).

Taken together, these sub-themes illustrate how therapy enabled caregivers to reinterpret behaviours through both trauma-informed and neurodevelopmental lenses. Rather than viewing behaviours as deliberate or oppositional, caregivers increasingly understood them as reflecting the combined effects of trauma, attachment disruption and PAE-related differences in brain functioning. This reframing appeared central to subsequent changes in parenting responses. Caregiver support therefore appears crucial in promoting therapeutic change, as opposed to adjuncts to child-focussed interventions.

### Theme 5: Redefining ‘Success’

Building on the changes described in the previous theme, caregivers described a shift in how therapeutic success was understood. As caregivers developed greater understanding of PAE and trauma, adapted their parenting approaches and became more responsive to their child’s needs, expectations of therapy also changed. Success was increasingly defined not by the resolution of difficulties, but by improved understanding, responsiveness and relational functioning.

#### Letting Go of Cure-Oriented Expectations

As caregivers developed a greater understanding of the enduring impact of PAE and trauma, many described a corresponding shift in their expectations of therapy. Caregivers began to realise that “the challenges don’t go away” (Participant 8), with one caregiver recognising “there’s always going to be a gap between where she should be and where she is” (Participant 4). Letting go of cure-orientated expectations was framed as a necessary shift that allowed caregivers to engage with their child’s needs more realistically and compassionately. This led to shifts in expectations from therapy, often focussing on “small wins” (Participant 8). Through this, caregivers recognised their role, with one reflecting “you know, [child]’s trauma is there, it’s something that we have to scaffold around. It’s always going to be there. But it’s how we kind of try and support [child] to deal with that” (Participant 9).

For families with an FASD diagnosis, caregivers recognised the impact is “lifelong” (Participant 3) and complex. Caregivers reflected on the likely need for long-term support, with one stating “the FASD isn’t going to get cured tomorrow. It’s going to be with her for life. So therefore, she’s going to need a serious level of support as she transitioned throughout her life” (Participant 8). Another caregiver felt that no amount of therapy would make a difference to their child “because his brain just doesn’t work that way” (Participant 10), emphasising the need for environmental changes and practical support instead. Caregivers therefore valued therapy not for resolving difficulties, but for supporting survival, coping and ongoing functioning, with caregivers wondering “I don’t know if we’d have got through it without them” (Participant 2).

#### Success as Understanding and Responsiveness

As expectations shifted, indicators of success also changed. Caregivers increasingly described success in terms of understanding their child, greater confidence in responding to challenges, reduced stress in everyday life and improved overall family functioning. One caregiver noted “because of our ability to parent her better, we’re more informed, so we’re able to minimise things” (Participant 8), while another described the impact as “life changing”, reflecting that “it’s made me a lot, my life a lot better, definitely. So I think in turn it’s made his life better” (Participant 6). Success was therefore constructed as parental competence rather than behavioural change. The adaptations described in theme 4 were consequently viewed as outcomes in their own right.

#### Living Better with Ongoing Complexity

Building on their revised expectations and increased confidence responding to challenges, caregivers increasingly described success in terms of sustaining family relationships and functioning despite ongoing difficulties. Caregivers described subtle but meaningful changes, including reduced conflict, increased emotional safety and improved parent-child relationships. One caregiver reflected that therapy had been “transformatory in terms of the relationship I now have with the children” (Participant 4), whilst another noted their child was “more affectionate and a lot more open” (Participant 1 A). For some families, these changes were experienced as particularly significant given the severity of previous difficulties. Caregivers described improved stability and reduced violence, with one stating “I think it all pretty much saved the placement” (Participant 5). These improvements were typically attributed to the changes in understanding, expectations and caregiving responses described in the previous theme.

However, caregivers emphasised that “change is not linear” (Participant 5), often describing the process of change as “cyclical” (Participant 2). In some cases, things deteriorated before improving. One caregiver explained “It’s like during therapy it’s all over the place because it’s throwing all his emotions everywhere, and then afterwards, when it all gets to be processed, he comes out a calmer person afterwards” (Participant 1B).

In two cases, therapy was perceived as making things worse or changing nothing, typically attributed to a perceived lack of therapeutic fit or relational safety. One caregiver noted “his behaviour has continued to get worse and decline” (Participant 7), whilst another reflected “that period of therapy triggered the worst period we’ve had with [child]. It got really sort of, we were in crisis really” (Participant 9).

Taken together, these sub-themes demonstrate how caregivers’ expectations of therapy were fundamentally shaped by their understanding of both PAE and trauma as enduring influences on development. Many participants described entering therapy hoping for substantial behavioural change or symptom reduction, but gradually shifted towards more realistic and compassionate expectations centred on adaptation, support and improved quality of life. Therapy was therefore repositioned from a means of curing difficulties to a means of helping families live alongside ongoing complexity.

## Discussion

This study explored caregivers’ experiences of family-based therapy for children with PAE and trauma, focusing on perceived benefits and challenges, children’s ability to engage, and caregivers’ understandings of therapeutic outcomes and factors influencing change. Types of therapeutic intervention varied across participants, with many families receiving a combination of approaches. Across five themes, therapeutic interventions were constructed as a relational, effortful and ongoing processes that support families to live with enduring complexity. Therapeutic change was primarily located within caregivers’ development of understanding, responsiveness and ability to adapt the caregiving environment, as opposed to within the child. Engagement emerged as negotiated and developmentally contingent, with the therapeutic relationship as a central component. Therapy was shaped by the perceived permanence of PAE and trauma, and by wider systemic constraints. The discussion below is organised around the research aims to situate these findings within existing literature and highlight their contribution.

### Perceived Benefits and Challenges

Therapeutic interventions were described as challenging, but worthwhile overall. Caregivers consistently constructed therapy as undertaken at times of crisis and exhaustion, reflecting the cumulative demands of caring for children with PAE and trauma. This aligns with existing literature documenting elevated caregiver burden within this population (Chamberlain et al., 2017). Caregivers emphasised the emotional and practical labour involved in engaging with therapeutic services, as has been previously documented (Burney et al., 2024), with systemic and structural constraints further shaping experiences (Reardon et al., 2017). Diagnostic recognition of FASD sometimes facilitated access to services and credibility within systems that often discounted trauma-based needs. However, professional understanding and access to diagnosis and therapeutic support was frequently framed as uneven and contingent, with caregivers describing support as a matter of luck rather than entitlement. This aligns with research documenting inequities in access to FASD diagnosis and specialist services in the UK (Mukherjee, 2019), and has been highlighted by other caregivers across the literature (Chamberlain et al., 2017). Similar challenges have been documented internationally, with caregivers in Canada, Australia and the United States reporting lengthy diagnostic pathways, difficulty accessing support and a need to advocate extensively for their children (Chamberlain et al., 2017; Hayes et al., 2023). Although the present study was conducted within the UK, the convergence of these findings with international literature suggests that many of the challenges surrounding diagnosis, access and caregiver burden may reflect broader systemic issues experienced by families affected by PAE across different service contexts. Navigating such systems requires substantial emotional and practical labour, compounding an already overburdened caregiving context.

Despite challenges, caregivers’ accounts emphasised the overall value of therapeutic interventions. This value was framed in the context of supporting their ability to cope, survive and function as opposed to resolving difficulties. Research has documented that children’s difficulties often remain following family-based interventions, yet caregivers are better able to tolerate these as a result of therapeutic support (Damen et al., 2021). This is further discussed in outcomes and factors influencing change.

However, caregiver accounts were not uniformly positive. Several participants described periods in which therapy appeared ineffective, poorly timed, or even destabilising. In some cases, therapy was perceived as exacerbating distress, particularly when children did not experience sufficient relational safety or the therapeutic approach was experienced as a a poor fit. These findings highlight the need for careful assessment, individualisation and ongoing review of therapeutic fit.

### Children’s Ability to Engage in Therapy

Despite the challenges accessing therapy, children and their caregivers were mostly able to engage in the therapeutic interventions received. From caregivers’ perspectives, children’s engagement emerged in response to relational safety, developmental readiness and therapeutic fit. However, caregivers resisted narrow driven definitions of engagement such as those based on attendance, verbal participation or compliance (Tetley et al., 2011) and instead described children engaging in diverse and subtle ways, including listening, observing, or selective participation. This is important to note given that many children’s services measure engagement through attendance and active participation (Gopalan et al., 2010). Furthermore, engagement was understood to fluctuate over time, sometimes on a week-by-week basis, and was shaped by developmental stage, emotional readiness and wider family circumstances, reflecting literature highlighting the impact of individual and contextual factors on children’s engagement in therapy (Levac, 2024). Periods of disengagement were often framed as situational or temporary, with caregivers anticipating that therapy might become more accessible or meaningful later in the child’s life. Timing was therefore illustrated as key.

Therapeutic fit played a central role in shaping engagement, with caregivers noting flexible, developmentally attuned and relationally responsive practice facilitated engagement, whilst misattunement and poor timing resulted in disengagement. This is consistent with trauma-informed and developmental literature highlighting the role of emotional regulation and relational safety in facilitating engagement (Bath, 2008; Perry, 2006). Play-based approaches were often positioned as preparatory, supporting parent-child attachment and therapeutic safety, enabling later verbal reflection. This reflects broader understandings of play as a medium for co-regulation and relationship-building (Landreth, 2012), particularly in the context of trauma (Aliannezhadi, 2025). As children developed, caregivers described shifts in what constituted a good therapeutic fit; talking-based approaches became more relevant, though engagement was often still supported by activities, movement, or sensory regulation. Sensory approaches were constructed as particularly helpful in supporting regulation, a key area of difficulty in children with neurodevelopmental differences and trauma (Cai et al., 2024; van der Kolk, 2003). These findings align with the therapeutic pyramid of need (Golding, 2020), suggesting that children require safety, positive relationships and co-regulation before being able to reflect and explore their trauma. This also mimics the neurosequential model of therapeutics (Perry, 2006), which recommends sequencing interventions from regulating activities to relational therapies and finally cognitive therapies. However, given the need for flexibility and adaptability highlighted, more integrative approaches, such as the neurocollaborative model may be most appropriate for this population (Purrington et al. 2025a, b).

### Potential Outcomes

Caregivers’ accounts challenged dominant assumptions regarding therapeutic outcomes by foregrounding relational and contextual change over children’s symptom reduction. In this sense, a key outcome of all therapeutic interventions appeared to be caregivers’ ability to cope, survive and function in the context of ongoing difficulties, as has been previously documented in family-based interventions (Damen et al., 2021). Caregivers emphasised outcomes such as improved understanding, increased responsiveness, relational stability and a greater capacity to tolerate complexity as opposed to observable behavioural change. This redefinition of success is particularly important given the enduring nature of FASD-related difficulties, aligning with critiques of outcome frameworks that prioritise symptom reduction over relational and contextual change (McLeod et al., 2024).

Caregivers described feeling more competent in their parenting ability, contributing to reduced escalation of difficulties, improved family functioning and relational safety. Research has highlighted the impact of parental empowerment on reducing parenting stress, thus positively impacting children’s wellbeing (Damen et al., 2021). These are particularly significant outcomes given the importance of stable, supportive caregiving environments for children with PAE (Jacobson et al., 2004; Streissguth et al., 2004) and trauma (Crouch et al., 2019). Importantly, change was described as cyclical and non-linear. Some families reported little improvement despite prolonged intervention, whilst others experienced temporary deterioration. These accounts suggest that outcome variability should be expected when working with children affected by both PAE and trauma and challenge simplistic assumptions regarding therapeutic effectiveness. Research has suggested that relational therapy can initially cause destabilisation as trust develops (Hughes & Baylin, 2012), potentially explaining this phenomenon.

### Factors Influencing Change

Caregivers strongly positioned themselves as the primary agents of therapeutic change, aligning with systemic and attachment-informed perspectives that locate change within caregiving relationships rather than within the child (Byng-Hall, 1999; O’Connor & Zeanah, 2003). Therapeutic interventions were described as most effective when they supported caregivers to reinterpret their child’s behaviour through trauma-informed and neurodevelopmental frameworks, shifting away from interpretations grounded in defiance or intentionality. This shift in narrative reduced frustration and blame, enabling caregivers to respond with greater empathy, attunement and containment. Such reframing is consistent with wider literature identifying meaning-making as a precursor to change (Kenny et al., 2017) and highlights the value of trauma-informed approaches (Milot et al., 2016). Caregiver involvement within sessions was therefore central to the therapeutic process. Family-based sessions provided a contained space for shared learning away from everyday pressures, whilst caregiver-only sessions offered emotional support, validation, reflection and opportunities to deepen understanding of PAE and trauma, which have been linked to increased parental competence and capacity (Davis et al., 2017; Liu et al., 2024). Together, these elements strengthened caregiver wellbeing, sustained engagement, and supported adaptations in parenting approaches. Caregivers began prioritising relational, regulatory, and child-specific strategies over traditional behavioural techniques, thus facilitating relational stability and sustained caregiving capacity. These findings reinforce caregiver-mediated change as a meaningful therapeutic outcome, and are consistent with literature emphasising the importance of caregiver involvement and support in promoting therapeutic engagement and success (Burney et al., 2024; Dowell & Ogles, 2010; Kenny et al., 2017), and the importance of systemic interventions for children who have experienced maltreatment (Carr, 2025).

Furthermore, the therapeutic relationship emerged as central in both facilitating engagement and as a mechanism of change, with caregivers emphasising trust, safety and continuity. This aligns with extensive literature highlighting the importance of the therapeutic alliance across therapy modalities (e.g. (Norcross & Lambert, 2019), including in child therapy (Nuñez et al., 2022). Caregivers frequently attributed therapeutic effectiveness to the individual therapist rather than specific models or techniques, thus challenging service structures that prioritise manualised, time-limited interventions and echoing critiques of standardisation within child mental health services (Georgiadis et al., 2020). Importantly, therapeutic relationships developed slowly over prolonged periods of time, likely explained by children’s experiences of past relational trauma (Bath, 2008). Once again, this aligns with the therapeutic pyramid of need (Golding, 2020) by suggesting safety and relationships must be established as a baseline to therapy. This further emphasises the need for long-term, flexible and consistent support for families with complex and enduring needs.

Validation was constructed as a particularly powerful process for both caregivers and children, both promoting therapeutic relationship and improving understanding. Caregivers highlighted the importance of the family feeling understood and accepted, aligning with previous research positioning validation as a tool for strengthening therapeutic relationships, fostering engagement and change (Nuñez et al., 2022). A diagnosis of FASD provided additional validation, as has been reported by other caregivers (Chamberlain et al., 2017). Research has suggested that diagnostic assessments can provide families with a framework for understanding their child’s difficulties, reducing self-blame and legitimising support needs (Hayes et al., 2023).

### Clinical and Service Implications

The findings suggest that family-based therapeutic approaches may hold particular value for children with prenatal alcohol exposure (PAE) and trauma. Participants’ accounts indicated that families may benefit from long-term, flexible models of support that prioritise relational safety and promote parental understanding and capacity. Sensory and play-based approaches were perceived by caregivers as especially helpful during the early years and may serve as a foundation for later talking-based interventions. These findings therefore suggest that integrating multiple therapeutic approaches could be beneficial. The findings also highlight the importance of recognising diverse forms of engagement and moving beyond narrow, compliance-based definitions of participation. Participants described caregiver-only sessions as valuable opportunities to support caregiver wellbeing and reflective capacity, suggesting that their routine inclusion may enhance therapeutic processes.

At a systemic level, the findings highlight the potential value of improved training in PAE and trauma-informed practice, alongside earlier and more equitable access to support. Participants’ experiences further suggest that caregiver-mediated change may represent an important indicator of progress, highlighting the need to consider whether existing outcome measures adequately capture relational and contextual change. Future research should explore the development of more sensitive and appropriate measures of therapeutic outcomes for this population.

Whilst the current study was conducted in the UK, the findings may have relevance beyond the national context. Similar barriers to diagnosis and access to specialised support have been documented internationally, particularly in Canada, Australia and the United States (Chamberlain et al., 2017; Hayes et al., 2023). The above implications may therefore be applicable more widely.

### Methodological Reflections and Limitations

The study is limited by a small sample of self-selected caregivers who had accessed therapy. Caregivers were mostly White British adoptive parents, with only one foster carer taking part, thus limiting generalisability. Due to the lack of formal diagnosis and documentation across participants, the level and timing of prenatal alcohol exposure could not be reliably established. Whilst recruitment was attempted across multiple sites in the UK, the majority of participants (*n* = 8) had accessed therapy from the same therapeutic service. Therapeutic approaches varied across participants, limiting claims about specific modalities. Recruitment predominantly took place using online methods such as via email, which may have limited accessibility. Perspectives of children and therapists were not included but may have provided additional insights. The dataset could have been strengthened through undertaking more interviews and incorporating greater diversity in the sample.

### Future Research

Further research is needed to enhance understanding of therapeutic approaches for families affected by PAE and trauma. Studies should move beyond child-centred and symptom-focused evaluations to examine the relational, contextual, and interpretive dimensions of therapeutic change. There is scope to develop outcome measures that more accurately capture shifts in parental understanding, responsiveness, and relational stability. Furthermore, Delphi methodology may offer a rigorous approach to developing integrative competency frameworks for practitioners working with this population. Such frameworks could help reduce inconsistency in practice while preserving the flexibility and adaptability that families in complex contexts require. Importantly, competency development should include caregivers and young people alongside clinicians and researchers, ensuring that relational and experiential dimensions of practice are embedded within professional standards. Future cross-national research could explore whether caregivers’ experiences of family-based trauma-informed interventions differ internationally.

## Conclusion

This study extends existing research by exploring how caregivers make sense of family-based therapeutic interventions for children affected by both prenatal alcohol exposure (PAE) and trauma. A key contribution of this study is its challenge to conventional outcome-focused models of therapy. Caregivers consistently described therapeutic benefit as arising through increased understanding, adaptation and relational responsiveness, as opposed to symptom reduction or recovery. Therapeutic change was primarily located within caregivers’ capacity to reinterpret behaviour, modify the caregiving environment and sustain supportive relationships in the context of enduring neurodevelopmental and trauma-related difficulties. Caregivers redefined success not as the resolution of difficulties, but as improved family functioning, relational stability and the ability to navigate ongoing complexity. This highlights the importance of family-based approaches that support caregivers alongside children and suggests that caregiver-mediated change may represent an important, yet often overlooked, therapeutic outcome for this population.

## Data Availability

The data that support the findings of this study are not publicly available due to the small sample size and the sensitive, qualitative nature of the data, which could risk identification of participants.
